# Photosynthetic Light Response Curve and Photosynthetic Performance of Cacao (*Theobroma cacao* L.) Genotypes Grown Under Full Sun Field Conditions

**DOI:** 10.3390/plants14233555

**Published:** 2025-11-21

**Authors:** Enilton Nascimento de Santana, Karin Tesch Kuhlcamp, Jeane Crasque, Basilio Cerri Neto, Vinicius de Souza Oliveira, Sara Dousseau-Arantes

**Affiliations:** Instituto Capixaba de Pesquisa, Assistência Técnica e Extensão Rural, Centro Regional de Desenvolvimento Rural—Norte, Linhares 29901-443, ES, Brazil

**Keywords:** high luminosity, hyperbola model, light curve, photosynthetic efficiency, solar radiation

## Abstract

The cultivation of *Theobroma cacao* L. in full sun conditions is expanding, but little is known about the physiological response of different genotypes under high irradiance in the field. This study evaluated the photosynthetic light response curve and physiological performance of eleven genotypes (BN-34, CSE-70, VT-05, FL-89, NSV-04, PS-13 19, NSV-410, CP-49, CEPEC-2002, VEM-20, and SJ-02) grown in full sun in Linhares, ES, Brazil. Parameters derived from gas exchange and hyperbolic modeling were used. Variability was observed among genotypes. The VT-05 genotype showed the best performance, with a high maximum CO_2_ assimilation rate (*P*_nmax_ = 10.92 µmol CO_2_ m^−2^ s^−1^), low dark respiration (R_d_ = 0.157 µmol CO_2_ m^−2^ s^−1^), reduced light compensation point (Γ = 2.73 µmol photons m^−2^ s^−1^), and high water use efficiency (WUE = 4.65 µmol CO_2_ mmol^−1^ H_2_O). Genotypes PS-13 19, CP-49, VEM-20, NSV-04, and CSE-70 also stood out with high WUE and instantaneous carbon use efficiency (EiC = µmol CO_2_ µmol^−1^ photons), indicating good performance under high light conditions. In contrast, BN-34 and NSV-10 showed low photosynthetic efficiency, lower EiC values, and higher vapor pressure deficits (Vpd = kPa), suggesting lower adaptability to full sun conditions.

## 1. Introduction

Cocoa (*Theobroma cacao* L.) holds significant global economic importance, with its beans serving as the main raw material for chocolate and its derivatives [1]. As an understory tree, it has traditionally been cultivated in shaded systems [2]. However, full sun cultivation has been expanding due to its higher yield potential, earlier harvests, and improved fruit quality [2,3,4,5]. Moreover, some genotypes have shown better adaptation to high light intensities [6]. The response to full sun varies among genotypes, necessitating the selection of more light-tolerant materials [2,7].

Studies have quantified the productivity gains associated with full-sun cacao cultivation, with yields ranging from approximately 1220 kg/ha in genotype CCN 10 to 2900 kg/ha in genotype CCN 51, depending on the genetic material and management practices adopted [5]. In addition to increased productivity, full-sun cultivation has been associated with improvements in the physical characteristics of fruits and beans, including larger dimensions, a higher number of beans per fruit, and greater dry mass [4]. However, the response to full sun is highly variable among genotypes, reinforcing the need for careful selection of materials more tolerant to high irradiance [2,7].

Exposure to high irradiance may initially enhance the photosynthetic rate and productive potential in cacao trees [5,6,8]. However, several studies indicate that intense light and elevated temperatures can lead to photoinhibition, reducing the physiological performance of plants [3,7,9]. In addition, the ability of cacao to adapt to high irradiance is influenced by genotypic factors [7,10].

The photosynthetic light response curve, which describes net CO_2_ assimilation (*P*_n_) as a function of irradiance (PPFD), is an essential tool for characterizing the photosynthetic performance and physiological plasticity of genotypes [7,8,11]. The derived from this curve, maximum photosynthetic rate (*P*_nmax_) and dark respiration rate (R_d_), are key indicators of genotypic adaptability to different light levels. Combined with gas exchange measurements and water use efficiency (WUE), these indicators help identify genotypes with higher productive potential under full sun conditions [7].

Studies have shown physiological variability among cacao genotypes under different light intensities [6,9], but *P*_n_/PPFD curves remain underexplored. Most studies involve only a few genotypes, are conducted under controlled conditions, and often use young plants [6,7,9]. In Colombia, Leiva-Rojas et al. [6] identified different light response ranges in three *T. cacao* genotypes, highlighting CCN51, which performed best between 500 and 1500 μmol photons m^−2^ s^−1^ and showed a *P*_nmax_ of 7.4 μmol CO_2_ m^−2^ s^−1^ in eight-year-old plants under full sun. Ortiz et al. [9] observed that high irradiance (100%) reduced photosynthetic efficiency, indicating photoinhibition. In Ghana, Mensah et al. [7] reported that cacao genotypes grown under full sun exhibited different photosynthetic compensation points and maximum efficiency, pointing to important genetic diversity for selection. Most of these studies were conducted in humid equatorial regions, limiting their applicability in tropical areas with higher solar radiation [8].

Physiological responses vary among genotypes across different environmental and seasonal conditions [12]. Considering cacao’s susceptibility to photoinhibition under high light intensity and the lack of studies on the photosynthetic response of genotypes in field conditions, this study hypothesizes that genotypes with higher photosynthetic efficiency and better water use are more adapted to full sun cultivation. Therefore, the aim of this study is to evaluate the photosynthetic light response curve and the photosynthetic performance of eleven cacao genotypes grown under full sun in field conditions.

## 2. Results

**Figure 1:**
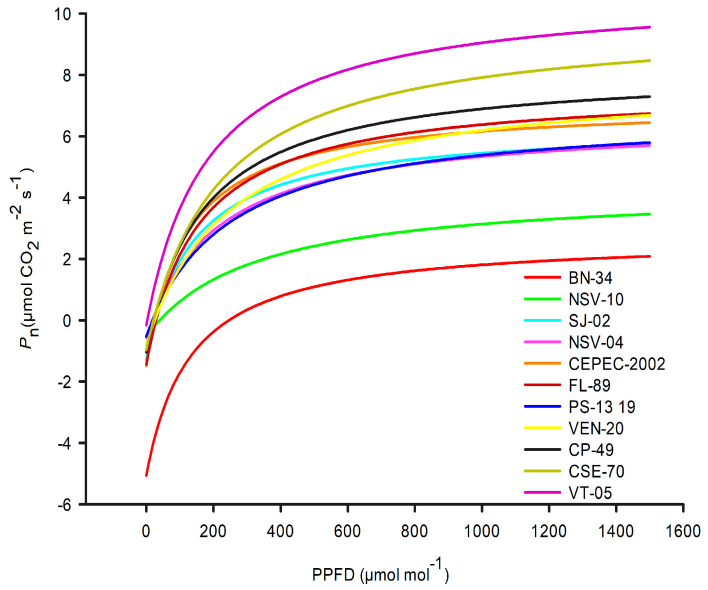
Net photosynthetic rate (*P*_n_) adjusted by the hyperbolic model for leaves of eleven cacao genotypes grown in full sun. The solid lines represent the adjusted *P*_n_/PPFD curves.

Net photosynthetic response curves as a function of photosynthetically active photon flux density were fitted for eleven cacao genotypes using a hyperbolic model (Figure 1). A progressive increase in the maximum CO_2_ assimilation rate was observed with increasing irradiance, reaching its peak at 1500 μmol photons m^−2^ s^−1^ in plants grown under full sun conditions.

Table 1 shows, in the hyperbolic model, the maximum assimilation rate ranged from 4.851 (NSV-10) to 10.924 (VT-05), with the highest values observed in VT-05 (10.924), followed by CSE-70 (10.759) and CP-49 (9.263). The lowest dark respiration rate was recorded in VT-05 (0.157 µmol CO_2_ m^−2^ s^−1^), while the highest was observed in BN-34 (5.060). The light compensation point varied among genotypes, with BN-34 showing the highest value (245.83). In contrast, the lowest values were found in VT-05 (2.73), NSV-04 (17.94), and PS-13 19 (18.57), suggesting greater photosynthetic efficiency under low-light conditions.

Among the variables evaluated in Table 2, only photosynthetic rate, stomatal conductance and transpiration did not show statistically significant differences among the genotypes. The internal CO_2_ concentration ranged from 360.96 to 303.35 µmol mol^−1^. Genotypes BN-34 (360.96), NSV-10 (350.53), CP-49 (340.70), CEPEC-2002 (347.77), SJ-02 (343.05), VT-05 (338.21), and NSV-04 (338.11) exhibited the highest internal CO_2_ concentration values. In contrast, genotypes PS-13 19 (327.52), FL-89 (325.98), CSE-70 (316.88), and VEN-20 (303.35) showed significantly lower values, with no statistical differences among them.

Similar results were observed for the internal to ambient CO_2_ concentration ratio, which ranged from 0.91 to 0.77 among genotypes. Genotypes BN-34 (0.91), NSV-10 (0.89), CP-49 (0.87), CEPEC-2002 (0.89), SJ-02 (0.87), VT-05 (0.87), and NSV-04 (0.86) showed the highest values, indicating a greater proportion of internal CO_2_ relative to the external environment, with no statistical differences among them. In contrast, genotypes PS-13 19 (0.83), VEN-20 (0.77), CSE-70 (0.81), and FL-89 (0.83) exhibited lower values. Water use efficiency varied widely among the evaluated genotypes. Genotype NSV-04 (6.01 µmol CO_2_ mmol^−1^ H_2_O) recorded the highest value. Following this, an intermediate group was formed by genotypes VT-05 (4.65), CP-49 (4.20), PS-13 19 (4.03), CSE-70 (3.89), and VEN-20 (3.86), followed by genotypes FL-89 (3.07), SJ-02 (3.14), CEPEC-2002 (2.59), and NSV-10 (2.26). The lowest water use efficiency was observed in genotype BN-34 (1.02).

Instant water use efficiency ranged from 13.36 to 47.48. Genotypes PS-13 19 (34.25), VEN-20 (47.48), CSE-70 (38.37), and FL-89 (34.25) presented the highest values, while the other genotypes showed no significant differences among them. Instant carboxylation efficiency ranged from 0.006 to 0.027 among genotypes. Genotypes BN-34 (0.006) and NSV-10 (0.012) showed the lowest values. In contrast, the other genotypes exhibited higher EiC values, with no statistically significant differences among them. The vapor pressure deficit at the leaf level and in the air showed the highest values in genotype BN-34 (1.88 kPa and 1.91 kPa, respectively). In contrast, the remaining genotypes exhibited significantly lower values, with no statistical differences among them. The analysis of Pearson correlation coefficients in Table 3 among physiological variables revealed that the maximum photosynthetic rate (*P*_nmax_) showed a significant positive correlation with carboxylation efficiency (EiC, 0.71 *), while exhibiting weak negative correlations with dark respiration (R_d_, −0.08 *) and the light compensation point (Γ, −0.2 *).

Dark respiration (R_d_) and Γ presented significant negative correlations with water use efficiency (WUE, −0.71 * and −0.69 *, respectively) and with EiC (−0.7 * and −0.76 *), and significant positive correlations with the vapor pressure deficit between the leaf and the air (VpdL and VpdA, 0.82–0.84 *). WUE showed a positive correlation with EiC (0.67 *) and negative correlations with VpdL and VpdA (−0.79 * and −0.77 *). The vapor pressure deficits between the leaf and the air (VpdL and VpdA) were extremely strongly correlated with each other (0.99 *), and instantaneous water use efficiency (EiWU) showed a positive correlation with VpdL (0.74 *).

## 3. Discussion

In response to the increasing demand for more efficient and resilient agricultural systems, it is essential to evaluate the photosynthetic adaptation of cacao genotypes with the aim of identifying materials better acclimated to high solar radiation incidence [13]. Existing studies largely focus on the characterization of a limited number of genotypes or fail to specify the evaluated materials, often being conducted under controlled conditions or high cloud cover typical of the Amazon region, which restricts the applicability of the results to full-sun cultivation systems [13].

Cacao’s response to light varies among genotypes, evidencing diversity in photosynthetic light response curves (*P*_n_/PPFD), a decisive factor for selecting materials more efficient under high irradiance [2,14].

The photosynthetic *P*_n_/PPFD curve is a key tool in this evaluation. Leiva-Rojas et al. [6] observed distinct genotype-specific responses to light intensity, corroborating the findings of the present study, where genotype-dependent variation was observed. This highlights the importance of genotypic analysis to guide management and material selection for cultivation in environments with high light incidence.

The maximum CO_2_ assimilation rate stood out as one of the most informative for differentiating genotypes. The VT-05 genotype proved most promising under full sun, exhibiting the highest maximum CO_2_ assimilation rate (*P*_nmax_, 10.92), combined with the lowest dark respiration rate (R_d_, 0.157) and the lowest light compensation point (Γ, 2.73), traits indicative of high photosynthetic efficiency even under intense irradiance. Additionally, VT-05 also demonstrates high productivity [15], reinforcing its potential for intensive cultivation systems and breeding programs.

These physiological advantages are particularly relevant from an agronomic perspective, as they can be translated into greater biomass accumulation, higher yield potential, and more efficient use of light in full-sun cultivation systems [4,5]. Previous studies have also linked high photosynthetic efficiency with improved pod and seed development in cacao [2,15], reinforcing the potential of VT-05 not only for intensive cultivation systems but also as a valuable genetic resource for breeding programs. The increase in photosynthetic rate observed in some genotypes may be associated with acclimation to high light intensity, favored by morphological traits such as canopy architecture, as reported by Benjamin et al. [5].

Genotypes such as CEPEC-2002 (8.55) and VEN-20 (8.71) also showed high performance, suggesting a greater capacity for carbon assimilation under high irradiance. Although we did not measure Rubisco activity directly, studies on cacao have used the maximum rate of Rubisco carboxylation (Vcₘₐₓ) and RuBP regeneration to demonstrate that genotypes well-acclimated to full sun can maintain or exhibit increases in these values compared to less tolerant genotypes [8,13].

Generally, low values of the light compensation point are desirable in plants cultivated under full sun because they allow earlier onset of net photosynthesis during the day and better use of diffuse light [14]. Genotypes such as VT-05, PS-13 19, CP-49, NSV-04, and CSE-70, which combine low light compensation points and low dark respiration rates with high maximum CO_2_ assimilation rates, exhibit great potential for breeding programs and intensive cultivation systems under high irradiance. Therefore, integrated evaluation of light compensation point, maximum CO_2_ assimilation rate, dark respiration, and correlation patterns among these traits is an effective strategy for selecting genotypes with greater photosynthetic efficiency and resilience in tropical systems.

It is important to consider plant age, as seedlings significantly reduce shade tolerance during their first year of growth, especially under exposed conditions. The light compensation point tends to increase as the canopy develops, requiring greater light quantity to balance photosynthesis and respiration [14]. Among species that survive under varying light levels, those with lower light compensation points during early stages, particularly under low light, exhibit faster growth, conferring a competitive advantage in variable environments. Additionally, Lahive et al. [2] emphasize that high respiration rates reduce net productivity, reinforcing the importance of selecting genotypes with low respiratory cost and high carbon assimilation.

Water use efficiency varied significantly among genotypes, revealing itself as an important indicator of adaptation to environments subject to water stress. This information is particularly relevant for the study region, where annual precipitation is approximately 1000 mm, as reported by Venancio et al. [16]. Genotypes originating from regions with lower rainfall exhibit less impact of drought on their water status [12]. Studies in low-precipitation regions can be beneficial for selecting suitable materials.

The genotypes NSV-04, VT-05, PS-13 19, and CP-49 showed the highest values of water use efficiency, suggesting efficient stomatal regulation and high carbon assimilation per unit of water lost [17]. These traits are desirable in regions with limited water availability or in intensive cultivation systems. Water use efficiency should be considered a key criterion in selecting drought-tolerant genotypes [6]. Genotypes with higher water use efficiency tend to exhibit better adaptation to intermittent radiation and variable field conditions, as further supported by the correlations showing negative relationships between water use efficiency and vapor pressure deficits.

Almeida et al. [12] demonstrated that physiological responses to drought vary among genotypes due to genetic variability, resulting in differences in physiological performance and seasonal adaptation.

The variable instant carboxylation efficiency also distinguished less efficient genotypes, such as BN-34 (0.006) and NSV-10 (0.012), indicating a low capacity to convert internal CO_2_ into assimilates. Conversely, genotypes with high instant carboxylation efficiency values suggest greater carboxylation activity, lower biochemical limitation, and reduced respiration-associated losses. These findings corroborate studies showing that cacao genotypes acclimated to intense light display higher photosynthetic rates and carbon use efficiency [13].

Internal CO_2_ concentration and the ratio between internal and external CO_2_ concentration complement physiological analysis. Genotypes such as BN-34 and NSV-10 presented high values of these parameters associated with low photosynthetic rates, indicating biochemical limitation in carbon fixation or excessive stomatal conductance without proportional assimilation.

In contrast, genotypes VT-05, PS-13 19, and CSE-70 exhibited low internal CO_2_ concentration and ratio values, reflecting higher Rubisco carboxylation efficiency [18]. BN-34 and NSV-10′s high internal CO_2_ concentration and ratio values suggest biochemical limitation or elevated stomatal conductance without proportional assimilation, a physiological pattern also observed under light or heat stress conditions [8]. On the other hand, genotypes PS-13 19, CSE-70, and VT-05 showed lower values for these indicators, suggesting more efficient use of available CO_2_, as also reported by Benjamin et al. [5] in genotypes grown under full sun.

Another notable physiological parameter was leaf vapor pressure deficit, which reflects the pressure difference between the leaf and the environment and directly influences stomatal opening. The BN-34 genotype presented elevated leaf vapor pressure deficit values (>1.8 kPa), suggesting higher water loss and reduced stomatal regulation capacity. This condition may impair water use efficiency, as evidenced by the data, and increase dehydration risk in environments with high evaporative demand [5]. According to Arévalo-Gardini et al. [19], elevated VpdL values are associated with reduced photosynthetic efficiency and decreased crop yield, making this parameter an important indicator in selecting genotypes more tolerant to water stress.

Several authors have identified genotypes with promising agronomic potential. For example, genotype PS-13 19 shows stability across different environments and good performance under high irradiance [5,8]. Genotype CP-49 stands out for its favorable leaf architecture and high photosynthetic capacity, being recommended for cultivation in areas with water deficit, along with Cepec-2002 [19,20].

Additionally, studies such as Lenno et al. [21] highlight that leaves exposed to intense light may exhibit photoinhibition, especially in tropical species like cacao. This reinforces the importance of selecting genotypes with a high capacity for acclimation to intense light and efficient energy dissipation mechanisms.

Some photosynthetic parameters did not show statistically significant differences among genotypes, which may be attributed to the limited number of samples and the measurement period, when light and temperature conditions were still moderate, reducing stress effects and physiological contrasts among genotypes. It is also worth noting that the curves, obtained from direct measurements of the photosynthetic rate and fitted using the hyperbolic model, allow the estimation of maximum photosynthesis and other derived indicators. These characteristics, extracted through mathematical fitting of the model, provide additional information that is not directly observable in individual measurements.

The importance of this study lies in its contribution to understanding the physiological and photosynthetic responses of cacao genotypes cultivated under full-sun conditions, a system increasingly adopted to enhance productivity. By identifying genotypes with greater photosynthetic efficiency, water use efficiency, and carbon assimilation capacity, this work provides essential information for breeding programs and for developing management strategies adapted to high-radiation and low-rainfall environments. Furthermore, the results strengthen the scientific basis for the sustainable intensification of cacao cultivation in tropical regions, supporting both environmental resilience and economic performance.

Finally, integrating the physiological factors analyzed here allows a more robust evaluation of genotypic suitability for full-sun cultivation. VT-05, PS-13 19, NSV-04, and CP-49 emerge as the most promising genotypes, combining desirable traits: high carbon assimilation, low respiration, high water use efficiency, low sensitivity to water stress, and high CO_2_ conversion efficiency. Conversely, BN-34 and NSV-10 showed significant physiological limitations and should be re-evaluated for use in intensive environments.

Therefore, selecting genotypes for full-sun production systems should consider multiple physiological indicators integrated with agronomic and climatic aspects. The combination of *P*_n_/PPFD curves, gas exchange, water use, and Pearson correlation patterns among key physiological traits is essential to guide breeding programs and define management strategies adapted to field realities.

## 4. Conclusions

The cacao genotypes PS-13 19, CP-49, VEN-20, NSV-04, and CSE-70 are promising for cultivation in regions with high solar irradiance and low water availability, due to their high photosynthetic efficiency, low dark respiration, and high water use efficiency.

The cacao genotypes BN-34 and NSV-10 presented significant physiological limitations, with high respiration rates, low photosynthetic efficiency, and greater apparent water stress, discouraging their use in intensive systems without specific management adjustments.

Although this study provides valuable insights, some limitations must be acknowledged. The evaluations were conducted at a single site and under specific environmental conditions, which may limit the generalization of the results. Future tests should include trials at multiple locations, seasonal monitoring, and long-term studies, including older plants at reproductive age, comparing performance in full-sun and shaded systems.

## 5. Materials and Methods

### 5.1. Study Area

The experiment was conducted at the Instituto Capixaba de Pesquisa Assistência Técnica e Extensão Rural (INCAPER), located in Linhares, Espírito Santo, Brazil (19°24′56″ S 40°04′41″ W, 11.51 m altitude). Climatic conditions were obtained from the automatic weather station of the Instituto Nacional de Meteorologia (INMET) during the experimental period, May 2019. The recorded average values were: maximum temperature 30.15 °C, minimum temperature 21.6 °C, mean temperature 25.88 °C; precipitation 9.55 mm; average relative humidity 78.7%; maximum radiation 2.486 MJ m^−2^.

### 5.2. Crop Description

**Figure 2:**
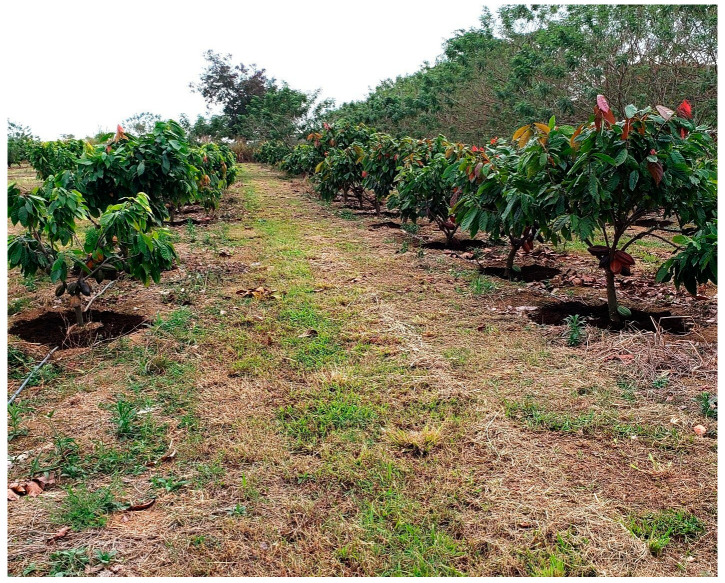
View of the experimental area with cacao genotypes grown under full sun conditions.

Eleven cacao (*Theobroma cacao* L.) genotypes sourced from the Comissão Executiva do Plano da Lavoura Cacaueira (CEPLAC), Bahia, Brazil, were evaluated. These genotypes were: BN-34, CSE-70, VT-05, FL-89, NSV-04, PS-13 19, NSV-10, CP-49, CEPEC-2002, VEM- 20, and SJ-02. Plants, at 1 year and 2 months of age and still in the vegetative stage, were cultivated under full sun with a spacing of 4.5 m between rows and 3.0 m between plants (Figure 2). During cultivation, the area received all necessary cultural practices, including irrigation, fertilization, mowing, hilling, pruning, and pest control.

### 5.3. Physiological Assessments of Gas Exchange

Gas exchange was measured using a portable infrared gas analyzer (IRGA LI–6400 XT, Li–Corporation, Lincoln, NE, USA). Measurements were performed between 8:00 and 10:00 under saturating light conditions with a photosynthetically active photon flux density of 1500 µmol photons m^−2^ s^−1^, at 25 °C and 500 ppm CO_2_.

The following parameters were measured: photosynthetic rate (*P*_n_, µmol CO_2_ m^−2^ s^−1^), stomatal conductance (gs, mol H_2_O m^−2^ s^−1^), transpiration (E, mmol H_2_O m^−2^ s^−1^), internal CO_2_ concentration (Ci, µmol mol^−1^), ratio between internal and external CO_2_ concentration (Ci/Ca, ppm/ppm), leaf vapor pressure deficit (VpdL, kPa), and vapor pressure deficit between leaf and air (VpdA, kPa). Based on these data, the following efficiency characteristics were calculated: water use efficiency (WUE = *P*_n_/E, µmol CO_2_ mmol^−1^ H_2_O), instantaneous water use efficiency (EiWU = *P*_n_/gs, µmol CO_2_ mol^−1^ H_2_O), and instantaneous carboxylation efficiency (EiC = *P*_n_/Ci, µmol CO_2_ µmol^−1^ CO_2_).

### 5.4. Model for Fitting Photosynthetic Light Response Curves and Parameters

Photosynthetic light response curves (*P*_n_/PPFD) were obtained by adjusting the light intensity in decreasing steps: 1500, 1200, 800, 500, 200, 100, 90, 80, 70, 65, 60, 55, 50, 45, 40, 35, 30, 25, 20, 15, 10, 5, 0 µmol photons m^−2^ s^−1^. Photosynthetic rate measurements were made at each light intensity, generating photosynthesis–light response curves for each genotype.

Curve fitting of *P*_n_/PPFD used the hyperbolic model described by Amarante [22] through the modified Michaelis-Menten modification. This response curve also allows calculation of the light compensation point in the dark (Γ), which corresponds to the light intensity at which net photosynthesis equals zero. Equation:

(1) $$P_{n}\;=\;-R_{d}\;+\;(P_{nmax}\;.\;PPFD)/(b\;+\;PPFD)$$

where *P*_n_ = photosynthetic rate (µmol CO_2_ m^−2^ s^−1^); *P*_nmax_ = maximum assimilation rate (µmol CO_2_ m^−2^ s^−1^); PPFD = light intensity (µmol photons m^−2^ s^−1^); R_d_ = dark respiration (µmol CO_2_ m^−2^ s^−1^); b = coefficient of the equation, related to the PPFD at which *P*_n_ reaches half of *P*_nmax_.

### 5.5. Experimental Design

The experiment was conducted in a randomized complete block design with 11 treatments, four replications, and 14 plants per plot. In each plot, three plants were selected, and one leaf per plant was analyzed.

### 5.6. Statistical Analysis

Data obtained from the IRGA were first tested for the assumptions of analysis of variance, with residual normality verified using the Shapiro–Wilk test. As the assumptions were met, ANOVA was applied, and means of the photosynthetic variables measured at a light intensity of 1500 µmol photons m^−2^ s^−1^ were compared using the Scott–Knott clustering test at a 5% probability level, which is considered appropriate for experiments with a large number of treatments. Statistical analyses were carried out in R software (version 4.4.2), using the ExpDes.pt (version 1.4.11) [23] and stats packages. Light response curves were fitted using a nonlinear hyperbolic model in SigmaPlot software (version 12.0). Pearson laboratory analyses were performed in the statistical program PAST [24] (version 1.0.0.0).

## Figures and Tables

**Table 1:** Values of the light–response curve equation: maximum photosynthetic rate (*P*_nmax,_ µmol CO_2_ m^−2^ s^−1^), dark respiration (R_d_, µmol CO_2_ m^−2^ s^−1^), light compensation point (Γ, µmol photons m^−2^ s^−1^), and coefficient of determination (R^2^) for eleven cacao genotypes.

Genotypes	Equation	*P*_nmax_	R_d_	Γ	R^2^
BN-34	$P_{n}=-5.060+(7.774.\;PPFD)/(131.873+PPFD)$	7.774	5.060	245.834	1.00
PS-13 19	$P_{n}=-0.523+(7.334\;.PPFD)/(241.331+PPFD)$	7.334	0.523	18.569	1.00
NSV-10	$P_{n}=-0.531+(4.851\;.PPFD)/(321.619+PPFD)$	4.851	0.531	39.601	1.00
CP-49	$P_{n}=-1.039+(9.263.\;PPFD)/(167.831+PPFD)$	9.263	1.039	21.224	1.00
CEPEC-2002	$P_{n}=-1.482+(8.555\;.\;PPFD)/(119.165+PPFD)$	8.555	1.482	24.970	1.00
VEN-20	$P_{n}=-0.803+(8.718\;.\;PPFD)/(246.046+PPFD)$	8.718	0.803	24.960	1.00
SJ-02	$P_{n}=-1.289+(7.663\;.PPFD)/(137.304+PPFD)$	7.663	1.289	27.781	1.00
CSE-70	$P_{n}=-0.964+(10.759\;.\;PPFD)/(211.820+PPFD)$	10.759	0.964	20.848	1.00
VT-05	$P_{n}=\;-0.157+(10.924\;.\;PPFD)/(186.923+PPFD)$	10.924	0.157	2.726	1.00
FL-89	$P_{n\;}=-1.437+(9.005\;.\;PPFD)/(151.853+PPFD)$	9.005	1.437	28.825	1.00
NSV-04	$P_{n}=-0.566+(7.133\;.\;PPFD)/(207.966+PPFD)$	7.133	0.566	17.944	1.00

**Table 2:** Physiological assessments of the gas exchange of leaves of eleven cacao genotypes grown in full sun. Photosynthetic rate (***P*_n_**, µmol CO_2_ m^−2^ s^−1^), stomatal conductance (gs, mol H_2_O m^−2^ s^−1^), transpiration (E, mmol H_2_O m^−2^ s^−1^), internal CO_2_ concentration (Ci, µmol mol^−1^), ratio between the internal and external concentration of CO_2_ (Ci/Ca, ppm/ppm), water use efficiency (WUE = ***P*_n_**/E, µmol CO_2_ mmol^−1^ H_2_O), instantaneous water use efficiency (EiWU = ***P*_n_**/gs, µmol CO_2_ mol^−1^ H_2_O), instantaneous carboxylation efficiency (EiC = ***P*_n_**/Ci, µmol CO_2_ µmol^−1^ CO_2_), vapor pressure deficit between the leaf (VpdL, kPa) and vapor pressure deficit between the leaf and the air (VpdA, kPa).

Genotypes	*P*_n_	gs	E	Ci	Ci/Ca	WUE	EiWU	EiC	VpdL	VpdA
BN-34	2.27 ^a^	0.14 ^a^	2.27 ^a^	360.96 ^a^	0.91 ^a^	1.02 ^d^	13.36 ^b^	0.006 ^b^	1.88 ^a^	1.91 ^a^
PS-13 19	6.95 ^a^	0.20 ^a^	1.73 ^a^	327.52 ^b^	0.83 ^b^	4.03 ^b^	34.25 ^a^	0.021 ^a^	0.89 ^b^	1.02 ^b^
NSV-10	4.19 ^a^	0.18 ^a^	1.74 ^a^	350.53 ^a^	0.89 ^a^	2.26 ^c^	21.96 ^b^	0.012 ^b^	1.07 ^b^	1.17 ^b^
CP-49	7.75 ^a^	0.29 ^a^	1.86 ^a^	340.70 ^a^	0.87 ^a^	4.20 ^b^	25.94 ^b^	0.023 ^a^	0.67 ^b^	0.82 ^b^
CEPEC-2002	6.56 ^a^	0.31 ^a^	2.52 ^a^	347.77 ^a^	0.89 ^a^	2.59 ^c^	21.66 ^b^	0.018 ^a^	0.90 ^b^	0.97 ^b^
VEN-20	7.51 ^a^	0.17 ^a^	2.04 ^a^	303.35 ^b^	0.77 ^b^	3.86 ^b^	47.48 ^a^	0.025 ^a^	1.27 ^b^	1.33 ^b^
SJ-02	5.73 ^a^	0.25 ^a^	1.86 ^a^	343.05 ^a^	0.87 ^a^	3.14 ^c^	25.82 ^b^	0.017 ^a^	0.85 ^b^	0.95 ^b^
CSE-70	8.79 ^a^	0.24 ^a^	2.33 ^a^	316.88 ^b^	0.81 ^b^	3.89 ^b^	38.37 ^a^	0.027 ^a^	1.04 ^b^	1.16 ^b^
VT-05	9.98 ^a^	0.39 ^a^	2.16 ^a^	338.21 ^a^	0.87 ^a^	4.65 ^b^	25.22 ^b^	0.026 ^a^	0.61 ^b^	0.76 ^b^
FL-89	6.69 ^a^	0.19 ^a^	2.09 ^a^	325.98 ^b^	0.83 ^b^	3.07 ^c^	34.25 ^a^	0.021 ^a^	1.20 ^b^	1.27 ^b^
NSV-04	6.17 ^a^	0.22 ^a^	1.15 ^a^	338.11 ^a^	0.86 ^a^	6.01 ^a^	29.67 ^b^	0.018 ^a^	0.58 ^b^	0.73 ^b^

Means followed by the same lowercase letter in the column do not differ significantly from each other, according to the Scott–Knott clustering test (*p* ≤ 0.05).

**Table 3:** Pearson correlation coefficients among physiological traits of full-sun cacao genotypes. Maximum photosynthetic rate (*P*_nmax,_ µmol CO_2_ m^−2^ s^−1^), dark respiration (R_d_, µmol CO_2_ m^−2^ s^−1^), light compensation point (Γ, µmol photons m^−2^ s^−1^), photosynthetic rate (*P*_n_, µmol CO_2_ m^−2^ s^−1^), water use efficiency (WUE = *P*_n_/E, µmol CO_2_ mmol^−1^ H_2_O), instantaneous water use efficiency (EiWU = *P*_n_/gs, µmol CO_2_ mol^−1^ H_2_O), instantaneous carboxylation efficiency (EiC = *P*_n_/Ci, µmol CO_2_ µmol^−1^ CO_2_), vapor pressure deficit between the leaf (VpdL, kPa) and vapor pressure deficit between the leaf and the air (VpdA, kPa).

Variables	*P*_nmax_	R_d_	Γ	WUE	EiWU	EiC	VpdL	VpdA
*P*_nmax_		−0.08 *	−0.2 *	0.29	0.3	0.71 *	−0.16	−0.15
R_d_			0.96 *	−0.71 *	−0.53	−0.7 *	0.82 *	0.82 *
Γ				−0.69 *	−0.55	−0.76 *	0.83 *	0.84 *
WUE					0.48	0.67 *	−0.79 *	−0.77 *
EiWU						0.74 *	−0.14	−0.14
EiC							−0.55	−0.55
VpdL								0.99 *
VpdA								

Note: * *p* < 0.05.

## Data Availability

The original contributions presented in this study are included in the article. Further inquiries can be directed to the corresponding author.

## Abbreviations

The following abbreviations are used in this manuscript:

*P*_nmax_	Maximum Rate of CO_2_ Assimilation
*P*_n_	Directory of open access journals
gs	Stomatal conductance
E	Transpiration
Ci	Internal CO_2_ concentration
Ci/Ca	Ratio between the internal and external concentration of CO_2_
WUE	Water use efficiency
EiWU	Instant water use efficiency
EiC	Instant carboxylation efficiency
VpdL	Vapor pressure deficit between the leaf
VpdA	Vapor pressure deficit between the leaf and the air
*P*_n_	Photosynthetic rate
R_d_	Dark respiration
Γ	Light compensation point
PPFD	Photosynthetically Active Radiation
